# Supplementary material for: The Usefulness of an Online Simplified Screening Questionnaire (SSQ) in Identifying Work-Related Cancers
Source: Healthcare (Basel). 2023 May 26;11(11):1563. doi: 10.3390/healthcare11111563 (PMC10252760; doi:10.3390/healthcare11111563)
Supplement: Supplementary file 1 [file healthcare-11-01563-s001.zip › healthcare-2271667-supplementary.pdf]

ANNEX:
Simplified Screening Questionnaire (SSQ)

NASAL CAVITIES AND PARANASAL SINUSES: Department Head and Neck / Skin and Melanoma
**Do you work or have you worked:**
1- In direct contact with wood dust, leather dust, cement dust, cereal dust, in direct contact with dust from the textile industry;<br>2- Asbestos dust (In contact with ASBESTO in civil construction, handling material containing asbestos fibers, such as roof tiles (Eternit type) and water tanks);<br>3- In the leather industry;<br>4- Direct contact with formaldehyde;<br>5- With RX equipment;<br>6- In agriculture handling Herbicide (Paraquat Gramoxone);<br>7- Furnaces (in general, from the chemical, coke and gas industries);<br>8- Nickel smelting;<br>9- Wood industry, sawmill and carpentry;<br>10-Isopropyl alcohol production industry;<br>11-Leather and footwear industry,<br>12-Textile industry;<br>13-Paper industry;<br>14-Oil industry;<br>15-Mechanical workshop as a car mechanic.
**ORAL CAVITY, PHARYNX AND LARYNX: Department Head and Neck / Skin and Melanoma**
**Do you work or have you worked:**
1-In direct contact with wood dust, leather dust, cement dust, cereal dust, in direct contact with dust from the textile industry;<br>2- Asbestos dust (In contact with ASBESTO in civil construction, handling material containing asbestos fibers, such as roof tiles (Eternit type) and water tanks);<br>3- In the leather industry;<br>4- Direct contact with formaldehyde;<br>5-Mechanical workshop as a car mechanic;<br>6-In agriculture handling pesticides;<br>7-Furnaces (in direct contact with coal soot);<br>8-Molder and modeler of glass;<br>9-Wood industry, sawmill and carpentry;<br>10-Pottery (potter);<br>11-Leather and footwear industry;<br>12-Textile industry;<br>13-Paper industry;<br>14-Oil industry;<br>15-Carpet installer;<br>16-Manufacture of rubber.
**MELANOMA: Department Head and Neck / Skin and Melanoma**
**Do you work or have you worked:**
1-In the production of herbicides, in the production of pesticides, in agriculture, handling pesticides and/or herbicides;<br>2-With chemical products, handling chemical products;<br>3-Airline pilot, Telephone operator, Telephone/telegraph installer;<br>4-Miner;<br>5-Business Site;<br>6-In outdoor occupancy; (mason, welder, salesman, rural worker, lifeguard, health workers, fisherman, traffic warden);<br>7-With RX equipment;
**SKIN CANCER: Department Head and Neck / Skin and Melanoma**
**Do you work or have you worked:**
1-In tar extraction,<br>2-In the manufacture of Creosote from wood;<br>3-Exposed to the sun, exposed to ultraviolet radiation, in outdoor occupancy; (mason, welder, salesman, rural worker, lifeguard, health workers, fisherman, traffic warden);<br>4-Ionizing radiation, high voltage equipment, RX equipment;<br>5- Mountaineering guide;<br>6-Miner;<br>7-Business Site;<br>8-In agriculture, handling pesticides and herbicides, handling chemical products.

STOMACH AND ESOPHAGUS: Upper Digestive Department
**Do you work or have you worked:**
1-With dust from civil construction (mason, bricklayer, etc);<br>2-Mining or with coal or metal dust;<br>3-Gas station attendant (or with fuel vapors);<br>4-In metallurgy or industry with steam from mineral oil;<br>5-Rural worker applying or handling herbicides;<br>6-Laundry with dry cleaning;<br>7-Carbon black;<br>8-In oil extraction;<br>9-In manufacturing in the electronics industry;<br>10-In the manufacture of leather;<br>11-Assistant or cleaning assistant.

LIVER: Upper Digestive Department
**Do you work or have you worked:**
1-Plastic industry;<br>2-Solvent industry;<br>3-Rural worker applying or handling herbicides or pesticides;<br>4-In copper production,<br>5-In the wood industry (with wood preservative),<br>6-In the manufacture of paints, plastics, rubber products, pigments, papers.

PANCREAS: Upper Digestive Department
**Do you work or have you worked:**
1-Plastic industry;<br>2-Solvent industry;<br>3-Rural worker applying or handling herbicides or pesticides;<br>4-In the production of copper;<br>5-In the wood industry (with wood preservative);<br>6-In production in the manufacture of paints, plastics, rubber products, pigments, papers.

MESOTHELIOMA: Thorax Department
**Do you work or have you worked:**
1- In asbestos mine;<br>2-In civil construction, handling material containing asbestos fibers, such as tiles (Eternit type) and water tanks;<br>3-In the production of fiber cement items (water tanks, tiles, thermal or acoustic insulation, etc.);<br>4-In the production of friction items (brake linings and pads, clutch disc, etc.);<br>5-In the production of special fabrics with mechanical and chemical resistance, thermal, electrical and waterproof insulation of any area;<br>6-In the production of sealing items;<br>7-In Chemical Industries, electro-electronic industries;<br>8-In refineries; metallurgy and steel, oil industry;<br>9-In the paper and cardboard industry;<br>10-Shipyards.

LUNG CANCER: Thorax Department
**Do you work or have you worked:**
1- Manufacture of pesticides with arsenic, in agriculture, applying or handling pesticides;<br>2- Miner; in the foundation of tin, copper, steel, iron, in metal mining and refining, in the production in the heavy metal or aluminum industry, in the manufacture of alloy components and metal coating,<br>3- In the manufacture of ceramics for electrical or electronic applications;<br>4- In the nuclear industry, in the medical device industry;<br>5- In the aircraft industry;<br>6- In the production of nickel-cadmium batteries;<br>7- In the production of refractory bricks;<br>8- In the production of paints and pigments,<br>9- In the glass industry, blowing or glass making;<br>10-In the production and tanning of leather;<br>11- In coal gasification, in coal mines, coal pitch, manganese and nickel, with dust from: coal, wood, rock/quartz and cement, radon, silica, uranium, ionizing radiation;<br>12- In the production of coke, in a coke oven;<br>13- Exposed to diesel combustion gases;<br>14- In the production of carbon black, or exposed to carbon black dust, tar, tobacco production;

15- Production or manufacture of shoes;
16- In production in the textile industry, weaver;
17- Hairdressers / Barbers using hair dye and / straightening with formaldehyde, in the production of dyes;
18- In the production of rubber, in the production of plastics;
19- In contact with ASBESTOS, in civil construction, handling materials that contain asbestos fibers, such as roof tiles (Eternit type) and water tanks;
20- In the Oil industry, cement, gypsum;
21- In the printing and paper industry.

LYMPHOID LEUKEMIA, MYELOID LEUKEMIA AND MYELODYSPLASIAS: Hematology Department

Do you work or have you worked:

1-Shoe industry;
2-Plastics industry;
3-Rubber industry;
4-Wood industry, with creosote, used in wood preservation;
5- Metallurgy and steelworks;
6-Oil refinery;
7-Gas station attendant;
8-In manufacturing or in contact with solvents (benzene);
9- In the manufacture of disinfectants, chemical sterilants;
10- With liquid used as a detergent for cleaning textiles and metals (Tetrachlorethylene);
11-Chromium (a metal used in metallurgy in dyes and inks);
12-Arsenic (used as a wood preservative, in the manufacture of insecticides and herbicides, in the manufacture of semiconductors and as a bleaching agent in the manufacture of glass);
13- Ethylene oxide (a gas used in sterilization);
14- In Agriculture, applying or handling pesticides, in the manufacture of pesticides;
15-Worker in the electrical sector (electromagnetic fields, with high voltage equipment);
16-Asbestos extraction mine; in civil construction, handling material that contains asbestos fibers, such as tiles and water tanks; in the production of fiber cement products (water tanks, tiles, thermal or acoustic insulation, etc.)
17-In the manufacture of friction products (brake linings and pads, clutch disc, etc.)
18- In the production of special fabrics that offer mechanical and chemical resistance, thermal, electrical and waterproof insulation in any area;
19- In the production of sealing products;
20-In electrical and electronic industries;
21-In the paper and cardboard industry.

NON-HODGKIN'S LYMPHOMA : Hematology Department

Do you work or have you worked:

1-In Agriculture, Rural Worker, applying or handling pesticides;
2-In the manufacture of synthetic rubber and plastics, including acrylic;
3-In the manufacture of disinfectants, chemical sterilants;
4- With dry cleaning;
5-In the manufacture of paints, plastics, rubber products, pigments, paper;
6-As a radio and telegraph operator;
7- In photographic laboratories;
8-As a galvanizer;
9-In the production of the leather-footwear industry;
10-In the rubber and plastic production industry,
11-In production in the ceramics and porcelain industry;
12-In the production of the timber industry;
13- In the production of the textile industry;
14-At the power plant.

MULTIPLE MYELOMA: Hematology Department

Do you work or have you worked:

1-In Agriculture, Rural Worker, applying or handling pesticides;
2-In the manufacture of RX equipment;
3-In clinics or hospitals as an RX technician, in contact with RX equipment;
4-In manufacturing or in contact with solvents (benzene);
5-In manufacturing in the cosmetic industry;
6-Metallurgy and steelworks (contact with waste metals such as mercury, lead and cadmium), handling heavy metal waste.

BREAST CANCER: Breast Department

Do you work or have you worked:

1-Plastic industry;

2-Solvent industry;<br>3-In Agriculture, Rural Worker, applying or handling pesticides;<br>4-Radio and telephone operator, flight attendant, Airline pilot, Telephone operator, Telephone/telegraph installer, high voltage equipment (electromagnetic fields);<br>5-Hairdresser (dye, definitive brush, volatile organic compounds),<br>6-Nurse or nursing assistant with a night shift;<br>7-In production in rubber production;<br>8-In the chemical industry;<br>9-In oil refinery;<br>10-In the manufacture of PVC.
**CENTRAL NERVOUS SYSTEM: Neurology Department**
**Do you work or have you worked:**
1-Rural worker applying or handling herbicides or pesticides;<br>2-In the production of copper, lead, mercury, mineral oil;<br>3-With wood preservative;<br>4-In the rubber and plastic, graphic and paper, petroleum, textile and pesticide industries;<br>5-With electrical and telephone services, nuclear power plant, radio and telephone operator, flight attendant, airplane pilot;<br>6-Oil refinery;<br>7-In the production and repair of motor vehicles.
**BLADDER: Urology Department**
**Do you work or have you worked:**
1-Production of aluminum;<br>2-Gasification of coal;<br>3-Iron Foundry, Steel Foundry, Metal Machining, Miner;<br>4- Coal pitch;<br>5-Tar, in tobacco production;<br>6-Carbon black dust;<br>7-Agriculture, applying or handling pesticides;<br>8-Production or manufacture of shoes;<br>9-In Textile Industry production, or as a weaver (jute and cotton spinning);<br>10-In the production in Industry or Manufacture of electronics,<br>11- Hairdressers/Barbers with hair dye; in the production of dyes and/or dyes;<br>12- In the production of rubber;<br>13- In the production of plastics;<br>14- In production in Chemical industry, Pharmaceutical industry;<br>15- Graphic operators;<br>16-In short;<br>17-Coke oven;<br>18-Exposure to diesel combustion gases;<br>19-Painter<br>20-Oil Industry;<br>21-With high voltage equipment, airline pilot, flight attendant, telephone operator, telephone/ telegraph installer, with electrical and telephone services, nuclear power plant, radio operator;<br>22- In clinics or hospitals as an RX technician, in contact with RX equipment;<br>23-Laundry, with dry cleaning
